# A Critical Investigation of Sick Euthyroid Syndrome in Chronic Heart Failure Patients: Addressing the Need for Accurate Thyroid Assessment

**DOI:** 10.7759/cureus.65985

**Published:** 2024-08-02

**Authors:** Madhulika L Mahashabde, Lokesh Kumar, Yash R Bhimani, Sai Krishna Reddy, Brugumalla V Nitendra Saketh, Siddharth S Gharge

**Affiliations:** 1 Department of Medicine, Dr. D. Y. Patil Medical College, Hospital and Research Centre, Dr. D. Y. Patil Vidyapeeth, Pune (Deemed to be University), Pune, IND

**Keywords:** chemiluminescent microparticle immunoassay (cmia), abbott architect ci8200, thyroid-stimulating hormone (tsh), reverse t3, boston criteria heart failure, statistical package: spss, chronic heart failure, non-thyroidal illness syndrome, sick euthyroid syndrome

## Abstract

Background

The body undergoes numerous metabolic changes during severe illness or physiological stress to protect itself by lowering metabolism and reducing overall demands. This evolutionary adaptation dates back to early human development, long before the advent of ICU facilities and advanced treatments. One such protective mechanism is Sick Euthyroid Syndrome (SES), also known as Non-thyroidal Illness Syndrome (NTIS). SES commonly occurs in critically ill patients and is frequently observed in conditions such as heart failure, chronic kidney disease, and severe sepsis. This syndrome is characterized by abnormal thyroid function tests in patients with acute or chronic systemic illnesses who do not have intrinsic thyroid disease. Typically, these patients exhibit low serum levels of triiodothyronine (T3), normal or low levels of thyroxine (T4), and normal or low thyroid-stimulating hormone (TSH) levels. SES is believed to be an adaptive response to illness, aimed at reducing the body's metabolic rate and conserving energy during severe physiological stress. This original article delves into SES's prevalence and clinical impact in these settings.

Materials and methods

The study aims to determine the prevalence of SES in patients with long-standing heart failure, elucidate the relationship between thyroid function and heart failure severity, and assess its impact on various hematological and clinical parameters. This observational, cross-sectional study was conducted at Dr. D. Y. Patil Medical College, Hospital and Research Centre, Pune, India, a 2011-bed hospital, over one and a half years. This study included 70 patients with chronic heart failure, aged 18 years and above, defined by a left ventricular ejection fraction of 40% or less and a Boston criteria score of 8 or more. Patients were excluded if they had a history of thyroid dysfunction, clinical sepsis, or were taking thyroid-affecting drugs.

Results

The study provides important insights into the prevalence and impact of SES in long-standing heart failure patients. It found that a significant 44.29% of these patients exhibited low T3 levels, highlighting the substantial occurrence of SES in this population. Additionally, the study revealed a negative correlation between N-terminal pro-b-type natriuretic peptide (NT-proBNP) levels, Boston score, and total T3, suggesting that as indicators of heart failure severity worsen, total T3 levels may decrease further. Another key finding is the high prevalence of anemia among heart failure patients, with a notable gender disparity: 92.11% of male patients were affected compared to 50% of female patients.

Conclusion

The study concluded that SES is significantly prevalent among long-standing heart failure patients, further indicating that thyroid suppression increases with the severity of heart failure. Recognizing SES can guide tailored treatments, prompting intensive monitoring and optimized heart failure management. Additionally, the study found a high prevalence of anemia, particularly among male patients, highlighting the need for gender-specific considerations in managing heart failure. These findings underscore the importance of routine thyroid function assessments and regular monitoring of anemia in heart failure patients. Future research should focus on improving clinical outcomes through comprehensive management of both thyroid function and anemia in these patients.

## Introduction

Sick Euthyroid Syndrome (SES), or Non-thyroidal Illness Syndrome (NTIS), is characterized by abnormal thyroid function tests despite the absence of intrinsic thyroid gland disease. This condition typically arises in the context of acute or chronic systemic illnesses such as severe infections, major surgery, trauma, heart failure, and renal failure. SES is marked by alterations in thyroid hormone levels, including triiodothyronine (T3), thyroxine (T4), and thyroid-stimulating hormone (TSH), reflecting the body's response to illness rather than a primary thyroid disorder. The prevalence of SES is notably high in critically ill patients, reaching up to 70% in intensive care units, 20-30% among heart failure patients, 40-60% in chronic kidney disease patients, and 40-70% in cases of sepsis [1,2].

The pathophysiology of NTIS involves a complex interplay of factors affecting thyroid hormone production, metabolism, and action. A key mechanism is the increased production of reverse T3 (rT3) and the decreased conversion of T4 to the more active T3, leading to low T3 levels and elevated rT3 levels. Inflammatory cytokines, such as interleukin-6 (IL-6) and tumor necrosis factor-alpha (TNF-α), significantly inhibit the activity of deiodinase enzymes responsible for converting T4 to T3 and alter thyroid hormone transport and metabolism [3]. Additionally, acute or chronic illnesses impair the conversion of T4 to T3 through changes in enzyme activity, modifications in thyroid hormone-binding proteins, effects of certain medications, and overall metabolic alterations [4]. Changes in the levels and binding affinity of thyroid hormone-binding proteins, such as thyroxine-binding globulin (TBG), transthyretin, and albumin, further affect the total and free levels of T3 and T4. 

Diagnosing SES primarily involves interpreting thyroid function tests within the context of an acute or chronic systemic illness. Key thyroid function tests include measurements of TSH, free thyroxine (FT4), free triiodothyronine (FT3), total T4, and total T3. Typically, TSH levels in SES are low, low-normal, or normal, and suppressed TSH levels without signs of hyperthyroidism may suggest SES. FT4 and total T4 levels in SES can be normal, low, or low-normal, indicating SES rather than primary hypothyroidism when low or low-normal FT4 is present with low TSH. FT3 and total T3 levels are usually low in SES; a low FT3 with normal or low FT4 and low or normal TSH is characteristic of the syndrome. Elevated rT3 levels can further support the diagnosis of SES, as there is an increased conversion of T4 to rT3 instead of T3 in SES. Follow-up thyroid function tests, once the underlying illness has resolved, are crucial to differentiate SES from primary thyroid disorders [5].

Cardiovascular disease, a leading cause of global mortality with over 17 million deaths annually, often involves ischemic heart disease [6]. Thyroid hormones significantly influence cardiovascular function, and thyroid dysfunction, affecting 9-15% of adults, can exacerbate heart failure symptoms. It has been discovered that 23.3% of patients who have had an acute myocardial infarction also have NTIS, enhancing the predictive value of in-hospital cardiovascular mortality in these patients [7]. Understanding the link between heart failure and thyroid dysfunction is crucial for effective patient care. SES, which is common among heart failure patients, is linked to poorer outcomes, including higher rates of mortality and morbidity. Identifying SES in these patients can help guide personalized treatments, ensuring closer monitoring and optimized management of heart failure.

This study aims to understand the prevalence of SES in heart failure patients. The goal is to understand its impact on clinical parameters and improve diagnosis and treatment, ultimately enhancing patient outcomes and quality of care.

## Materials and methods

Study design and ethical approval

This observational, cross-sectional study was conducted at Dr. D. Y. Patil Medical College, Hospital and Research Centre, Pune, India, over one and a half years, from November 2022 to April 2024. Ethical approval for this study was obtained from the Institutional Ethics Sub-committee, with the approval letter number I.E.S.C/274/2022, before the start of the study.

Sample size calculation

Considering the prevalence of SES in patients with chronic heart failure to be 39.6%, as determined by Kemnang et al. (2021) in a study conducted at Yaoundé Central Hospital in Cameroon, the minimum sample size was calculated to be 68 [8]. This calculation, performed using WINPEPI version 11.65 (Brixton Health, London, UK), assumed an acceptable precision of 12% and a 95% confidence interval. Ultimately, 70 patients were recruited to ensure more robust and reliable results.

Method of study

**Figure 1:**
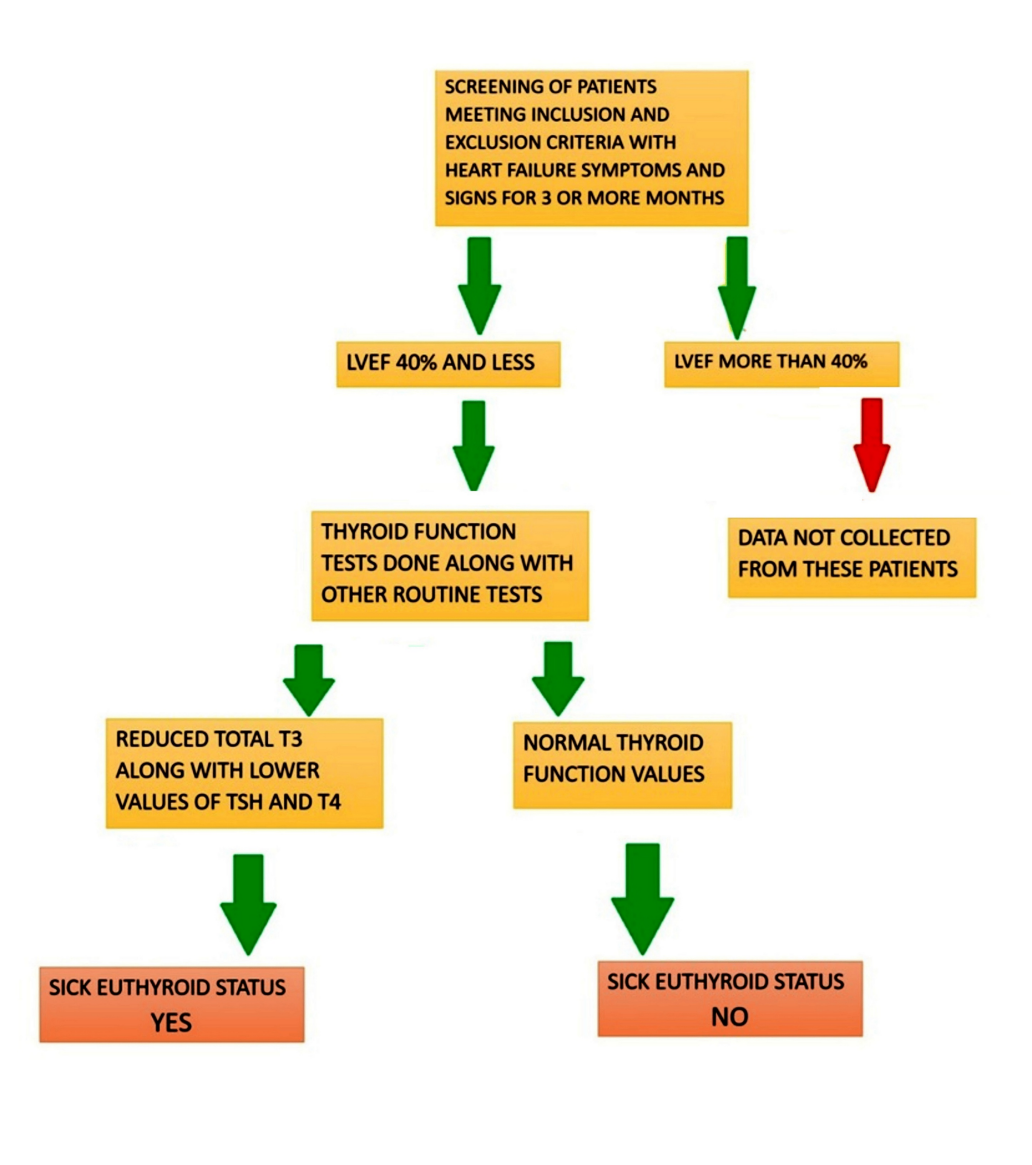
Outline for assessment of sick euthyroid status in chronic heart failure patients LVEF: Left ventricular ejection fraction; TSH: Thyroid stimulating hormone; T4: Thyroxine (total T4); T3: Triiodothyronine (total T3)

The screening of patients was conducted according to the flowchart (Figure 1). Patients were selected based on specific inclusion and exclusion criteria. Those included in the study were over 18 years old, had been experiencing symptoms of chronic heart failure for at least three months, and had a left ventricular ejection fraction (LVEF) of 40% or less. Additionally, only patients with a Boston score of 8 or higher were considered, ensuring the inclusion of individuals with significant heart failure severity. Patients with a history of, or clinical or laboratory evidence of, hypothyroidism or hyperthyroidism, clinical evidence of sepsis, or those taking medications that affect thyroid status (such as amiodarone) were excluded from the study. The study recruited 70 patients, explained the procedure and purpose to them, and obtained written informed consent from a family member. Physical examinations and necessary investigations were then performed.

Boston score in heart failure assessment

The Boston score/criteria are used to classify heart failure severity based on history, physical examination, and chest X-ray findings. Scores range from 0 to 12 and indicate the likelihood of heart failure (Table 1) [9,10].

**Table 1:** Boston score calculation criteria Each of the three categories - history, physical examination, and chest X-ray findings - can contribute a maximum of 4 points, resulting in a total possible Boston score of 12. A total score of 0-4 indicates a low likelihood of heart failure, 5-7 suggests an intermediate likelihood, and 8-12 denotes a high likelihood of heart failure.

Category	Criteria	Points
History		
Dyspnea on exertion	No dyspnea	0
	Dyspnea on moderate exertion	1
	Dyspnea on minimal exertion	2
	Dyspnea at rest	3
Orthopnea	None	0
	Uses one pillow	1
	Uses two pillows	2
	Uses three or more pillows	3
Paroxysmal nocturnal dyspnea	None	0
	Present	1
Physical examination		
Neck veins	Normal	0
	Jugular venous distension halfway up the neck	1
	Jugular venous distension up to the angle of the jaw	2
Rales	None	0
	Basilar rales	1
	More than basilar rales	2
Third heart sound (S3)	Absent	0
	Present	1
Edema	None	0
	Ankle edema	1
	Edema above the ankles	2
Chest X-ray findings		
Alveolar edema	None	0
	Present	1
Interstitial edema	None	0
	Present	1
Pleural effusion	None	0
	Present	1

Radiological and laboratory criteria for diagnosis

Ejection fraction (EF) is a crucial indicator of heart function and is commonly used to diagnose and monitor heart conditions such as heart failure. An EF of 55% to 70% is considered normal, while an EF below 40% may indicate heart failure or cardiomyopathy [11]. LVEF was measured using a 2D echocardiogram. Thyroid function tests, including TSH, FT4, FT3, total T4, and total T3, were measured using the Abbott Chemiluminescent Microparticle Immunoassay (CMIA) kit for advanced immunoassay testing [12]. Measurements were conducted with the Abbott Architect ci8200 Immunoassay (Abbott Laboratories, Abbott Park, IL, USA) and Clinical Chemistry Analyzer. Results were interpreted after statistical analysis.

Data analysis

The data were entered into a Microsoft Excel 2010 spreadsheet (Microsoft® Corp., Redmond, WA, USA) and imported into IBM SPSS Statistics GradPack, Version 29 (Released 2023; IBM Corp., Armonk, NY, USA) [13]. Descriptive statistics were calculated, including standard deviation, averages, and percentages. The statistical significance of parametric and nonparametric data distributions was evaluated using Pearson's correlation coefficient and the Chi-square test. A p-value of less than 0.05 and a 95% confidence interval were considered statistically significant. The final step of the study involves conducting a thorough discussion, summarizing key findings, and drawing conclusions based on the collected data.

## Results

Observation of symptoms in study group

**Figure 2:**
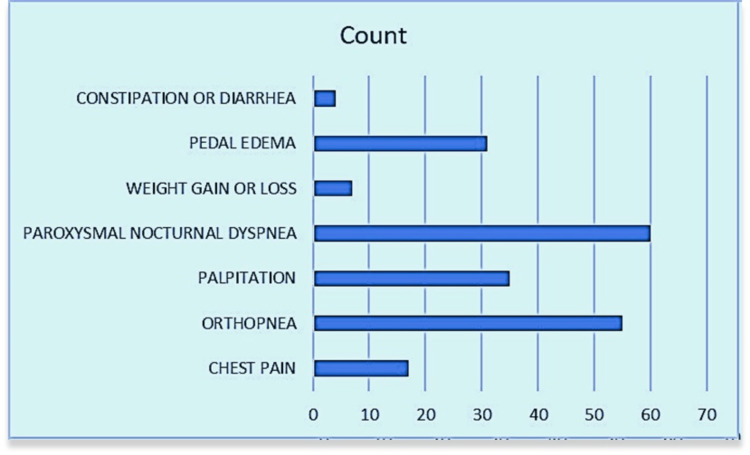
Bar diagram showing the number of patients with various symptoms (n = 70) X-axis: Number of patients; Y-axis: Symptoms observed in patients; n: Total number of patients

Out of 70 patients, 17 (24.3%) experienced chest pain, while 55 (78.6%) had orthopnea. Palpitations were reported by 35 patients (50%), and paroxysmal nocturnal dyspnea was the most common symptom, affecting 60 patients (85.7%). Weight changes were noted in seven patients (10%), and pedal edema was observed in 31 patients (44.3%). Constipation or diarrhea was the least common symptom, reported by four patients (5.7%). This distribution highlights the prevalence of paroxysmal nocturnal dyspnea and orthopnea within the patient population (Figure 2).

Observation of hemoglobin level in patients

**Figure 3:**
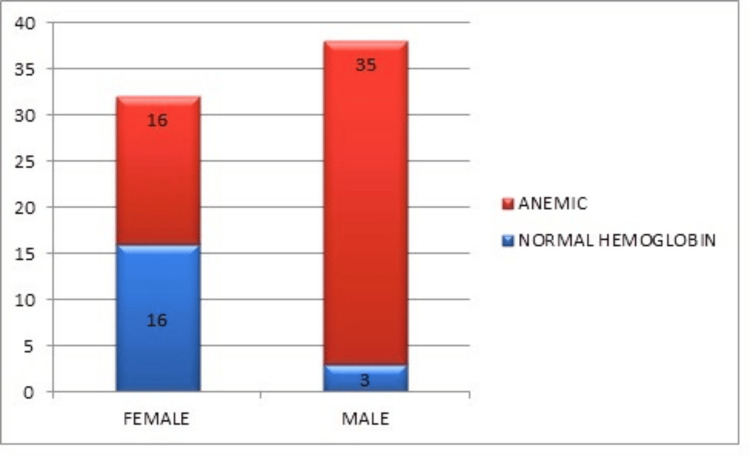
Distribution of hemoglobin levels by gender in patients (n = 70) n: Total number of patients

In this study of 70 patients, the mean hemoglobin level was 10.92 g/dL, with a median of 11.0 g/dL, indicating mild anemia. The mode was 12.0 g/dL, highlighting anemia's prevalence in chronic heart failure patients, which can worsen symptoms and complicate heart failure management. Anemia was significantly more prevalent among male patients, with 92.11% showing low hemoglobin levels compared to 50.00% of female patients. The mean hemoglobin level for female patients is 11.028 g/dL, slightly higher and more variable than that of males, who have a mean of 10.845 g/dL. Males have a lower mean, indicating a higher prevalence of anemia among them (Figure 3).

Observation of total T3 levels in study group

**Figure 4:**
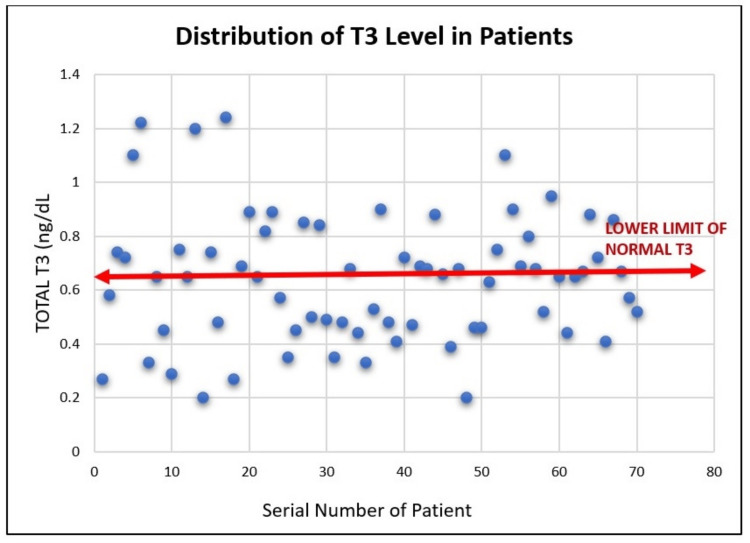
Distribution of T3 levels in patients (scatter diagram) The red line denotes the lower limit of the normal range of T3 (0.64 to 1.52 ng/dL). T3: Triiodothyronine, ng/dL: Nanograms per deciliter

In this study, 31 out of 70 patients (44.29%) exhibited low T3 levels despite normal TSH and T4 levels, indicating a significant incidence of SES or NTIS. The remaining 39 patients (55.71%) had normal T3 levels (Figure 4). This finding underscores the prevalence of thyroid function abnormalities in critically ill patients and the need for monitoring and managing these parameters to improve outcomes.

Observation of renal function tests in study group

In this study, 39 out of 70 patients (55.71%) exhibited elevated renal function tests, indicating impaired kidney function. This significant incidence underscores the prevalence of kidney-related issues among the study population, often reflected by increased serum creatinine and blood urea nitrogen (BUN) levels (Table 2).

**Table 2:** Distribution of renal function tests in patients

Renal function category	Number of patients	Percentage (%)
Deranged renal function	39	55.71%
Normal renal function	31	44.29%
Total	70	100%

Observation of N-terminal pro-b-type natriuretic peptide (NT-proBNP) levels in study group

In this study of heart failure patients, a staggering 97.92% of the 48 tested patients exhibited raised NT-proBNP levels, with 45 out of 48 (93.75%) showing grossly elevated levels (Table 3). Elevated NT-proBNP levels are a crucial biomarker for heart failure, indicating increased cardiac stress and ventricular dysfunction.

**Table 3:** Observation of NT-proBNP levels in the study group NT-proBNP: N-terminal pro-B-type natriuretic peptide

NT-proBNP level category	Number of patients	Percentage (%)
Grossly raised NT-proBNP levels	45	93.75%
Mild to moderately raised NT-proBNP levels	3	6.25%
Normal NT-proBNP levels	1	2.08%
Total	48	100%

Prevalence of sick euthyroid status in study group

**Figure 5:**
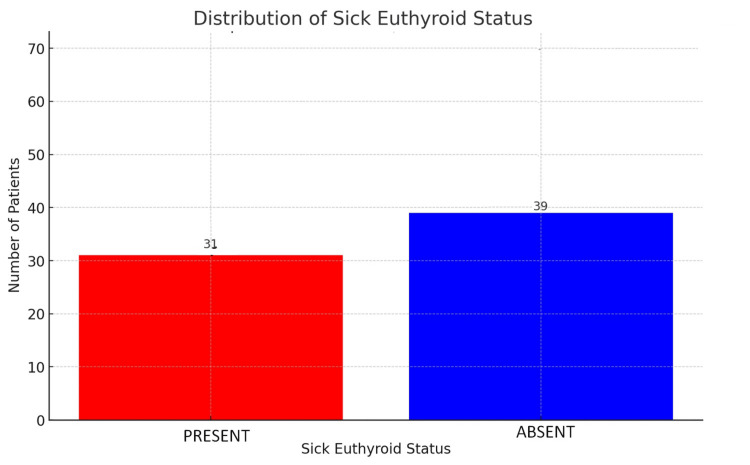
Distribution of sick euthyroid status in the study group

In this study of heart failure patients, the presence of SES was identified in 31 out of 70 patients (44.29%). The remaining 39 patients (55.71%) did not exhibit sick euthyroid status. The relatively high incidence of SES in this cohort underscores the complex interplay between thyroid function and cardiac health, particularly in critically ill patients (Figure 5).

Correlation analysis between dyspnea NYHA class, hemoglobin, jugular venous pressure, creatinine, and troponin I

The study reveals a statistically significant moderate negative correlation between hemoglobin and creatinine (Pearson correlation coefficient of -0.403, p-value of 0.001), suggesting that higher creatinine levels are associated with lower hemoglobin levels, which is often observed in chronic kidney disease. The covariance of -2.112 further supports this relationship. Other correlations are weak and non-significant (Table 4).

**Table 4:** Correlation analysis between dyspnea NYHA class, hemoglobin, jugular venous pressure, creatinine, and troponin I The table shows the Pearson correlation coefficients with their corresponding p-values in parentheses. Asterisks (*) denote statistically significant correlations, where p < 0.05. JVP: Jugular venous pressure, NYHA: New York Heart Association Classification, cm: Centimeters, g/dL: Grams per deciliter, mg/dL: Milligrams per deciliter

	Dyspnea NYHA class	Hemoglobin (g/dL)	JVP (cm of water)	Creatinine (mg/dL)	Troponin I
Dyspnea NYHA class	1	0.016 (0.895)	0.083 (0.494)	-0.039 (0.748)	-0.052 (0.703)
Hemoglobin (g/dL)		1	-0.213 (0.077)	-0.403 (0.001)*	-0.062 (0.646)
JVP (cm of water)			1	0.173 (0.153)	-0.078 (0.562)
Creatinine (mg/dL)				1	-0.058 (0.668)
Troponin I					1

Correlation analysis between NT-proBNP, total T3 levels, and Boston score

This analysis examines the relationships between NT-proBNP (pg/mL), total T3 (ng/dL), and the Boston score. A significant negative correlation is observed between NT-proBNP and total T3, with a Pearson correlation coefficient of -0.417 and a p-value of 0.003. This strong inverse relationship suggests that higher NT-proBNP levels are associated with lower total T3 levels. NT-proBNP is a marker of heart failure severity, while total T3 is a thyroid hormone, indicating that, as heart failure severity increases, T3 may be suppressed. The covariance value of -1445.281 further supports this negative association.

Total T3 and the Boston score also show a significant negative correlation, with a Pearson correlation coefficient of -0.239 and a p-value of 0.047. This significant inverse relationship suggests that higher Boston scores are associated with lower total T3 levels, implying that worsening heart failure may be linked to reduced thyroid function. In conclusion, the analysis reveals significant negative correlations between NT-proBNP and total T3, and between Boston score and total T3, suggesting that thyroid function may be suppressed as heart failure severity increases (Table 5).

**Table 5:** Correlation analysis between NT-proBNP, total T3 levels, and Boston score The table shows the Pearson correlation coefficients with their corresponding p-values in parentheses. Asterisks (*) denote statistically significant correlations, where p < 0.05. NT-proBNP: N-terminal pro-B-type natriuretic peptide; T3: Triiodothyronine; pg/mL: Picograms per milliliter; ng/dL: Nanograms per deciliter

	NT-proBNP (pg/mL)	Total T3 (ng/dL)	Boston score
NT-proBNP (pg/mL)	1	-0.417 (0.003)*	-0.01 (0.944)
Total T3 (ng/dL)		1	-0.239 (0.047)*
Boston score			1

Comparison between female and male patients for various variables

The comparison between female and male patients reveals that females tend to be slightly older, with a mean age of 59.41 years compared to 55.95 years for males, and have significantly higher hemoglobin levels (11.028 g/dL vs. 10.845 g/dL). Additionally, females exhibit higher mean fasting total cholesterol levels (152.52 mg/dL vs. 134.83 mg/dL), while males show higher NT-proBNP levels (19094.52 pg/mL vs. 15244.48 pg/mL), indicating greater cardiac stress in males. The rest of the findings show no significant differences between the two genders (Table 6).

**Table 6:** Comparison between female and male patients for various variables The table shows the mean value with standard deviation in parentheses for each variable. LV: Left ventricular; N: Number of patients; cm: Centimeters; g/dL: Grams per deciliter; mg/dL: Milligrams per deciliter; ng/dL: Nanograms per deciliter; pg/mL: Picograms per milliliter; NT-proBNP: N-terminal pro-B-type natriuretic peptide

Variable	Female (N = 32)	Male (N = 38)
Age of patients (years)	59.41 (13.254)	55.95 (15.488)
JVP (cm of water)	7.91 (0.734)	7.84 (0.916)
Hemoglobin (g/dL)	11.028 (2.2388)	10.845 (2.0761)
Total T3 (ng/dL)	0.5844 (0.19171)	0.6874 (0.26421)
Fasting total cholesterol (mg/dL)	152.52 (62.223)	134.83 (45.481)
Creatinine (mg/dL)	2.5950 (2.65963)	2.9337 (2.28136)
NT-proBNP (pg/mL)	15244.48 (15460.972)	19094.52 (15919.027)
LV ejection fraction (%)	32.81 (6.468)	30.66 (6.894)
Boston score	10.19 (1.061)	10.21 (1.212)

Comparison between groups with and without sick euthyroid status

Patients with sick euthyroid status tend to be older (59.90 years vs. 55.64 years) and have higher pulse rates (111.81 bpm vs. 103.77 bpm) compared to those without sick euthyroid status. They also have lower hemoglobin levels (10.66 g/dL vs. 11.14 g/dL) and higher creatinine levels (3.27 mg/dL vs. 2.39 mg/dL), indicating more severe comorbidities and potential renal impairment. Additionally, SES patients show significantly higher NT-proBNP levels (22637.91 pg/mL vs. 12292.56 pg/mL), suggesting greater cardiac stress. SES patients also have a slightly lower LVEF (30.97% vs. 32.18%) and a higher Boston score (10.39 vs. 10.05), reflecting worse heart failure severity (Table 7).

**Table 7:** Comparison between groups of positive and negative sick euthyroid status The table shows the mean value with standard deviation in parentheses for each variable. N: Number of patients; g/dL: Grams per deciliter; mg/dL: Milligrams per deciliter; pg/mL: Picograms per milliliter; NT-proBNP: N-terminal pro-B-type natriuretic peptide; bpm: Beats per minute; LV: Left ventricle

Parameter	Negative sick euthyroid status (N = 39)	Positive sick euthyroid status (N = 31)
Age of patients (years)	55.64 (13.84)	59.90 (15.21)
Pulse rate (bpm)	103.77 (12.78)	111.81 (15.01)
Hemoglobin (g/dL)	11.14 (2.25)	10.66 (1.99)
Fasting total cholesterol (mg/dL)	148.77 (52.88)	132.55 (55.60)
Creatinine (mg/dL)	2.39 (2.40)	3.27 (2.46)
NT-proBNP (pg/mL)	12292.56 (14810.64)	22637.91 (15033.06)
LV ejection fraction (%)	32.18 (6.57)	30.97 (7.00)
Boston score	10.05 (1.12)	10.39 (1.15)

## Discussion

The present study aimed to investigate the prevalence and relationship between thyroid function and heart failure severity in a cohort of 70 heart failure patients, focusing on SES. Notably, the study identified that a significant proportion of these patients exhibit characteristics associated with SES, aligning with findings from previous research that emphasize the prevalence of thyroid dysfunction among heart failure patients. The results showed that 44.29% of patients had low T3 levels despite normal TSH and T4 levels, highlighting the complex interplay between heart failure and thyroid function. This prevalence is consistent with earlier studies, such as those by Iervasi et al. (2003) [14] and Friberg et al. (2002) [15], which also reported a high incidence of SES in heart failure patients.

Interestingly, the study found a significant negative correlation between NT-proBNP and total T3 levels (r = -0.417, p = 0.003), suggesting that higher NT-proBNP levels, indicative of more severe heart failure, are associated with lower total T3 levels. This relationship underscores the role of thyroid function in the pathophysiology of heart failure, as worsening heart failure appears to suppress thyroid hormone levels. This finding is consistent with Fommei and Iervasi's (2002) [16] work, which demonstrated a similar inverse relationship between heart failure severity and thyroid hormone levels. Furthermore, the negative correlation between total T3 and the Boston score (r = -0.239, p = 0.047) reinforces the link between heart failure severity and thyroid dysfunction. This indicates that higher heart failure severity scores are associated with lower total T3 levels.

The study revealed significant insights into blood pressure and other clinical parameters among heart failure patients. Among the blood pressure readings, 42.86% were within the normal category, 7.14% were classified as elevated, 17.14% fell into Hypertension Stage 1, and 32.86% were categorized as Hypertension Stage 2, indicating severe hypertension. When comparing these findings with the study by McDonagh et al. (2021) [17] titled 'Epidemiology of Heart Failure,' several similarities and differences are noted. Their study found that approximately 40-45% of heart failure patients had hypertension, which closely aligns with the current study's findings of 17.14% of patients with Hypertension Stage 1 and 32.86% with Hypertension Stage 2. This consistency highlights the importance of continuous blood pressure monitoring and management in heart failure patients to mitigate severe hypertension and related complications. Additionally, McDonagh et al.'s (2021) study reported a similar gender distribution, with approximately 55% male and 45% female patients, predominantly older adults over 60 years old. Symptom prevalence in McDonagh et al.'s (2021) study included 70-80% experiencing dyspnea, with orthopnea and paroxysmal nocturnal dyspnea affecting 60-80% of patients, aligning closely with the current findings.

Renal function tests in the current study revealed that 55.71% of patients had elevated values, indicating impaired kidney function, with mean urea and creatinine levels at 67.57 mg/dL and 2.7789 mg/dL, respectively. These findings suggest renal insufficiency due to reduced cardiac output in heart failure patients. This is consistent with the findings from Heywood et al. (2007) [18], who reported a high prevalence of renal dysfunction in heart failure patients.

Several important differences emerged when comparing clinical characteristics between female and male patients. Female patients were slightly older on average (59.41 years) compared to male patients (55.95 years), which could be attributed to gender-related differences in disease progression and longevity. Hemoglobin levels were also higher in females (11.028 g/dL) than in males (10.845 g/dL), suggesting that anemia is more prevalent in male heart failure patients. This finding corroborates the study by Kajimoto et al. (2015) [19], which reported a higher prevalence of anemia in male heart failure patients. Additionally, NT-proBNP levels were significantly higher in males, indicating greater cardiac stress - a result that aligns with the findings of Alehagen and Dahlström (2009) [20], who observed higher NT-proBNP levels in male patients with severe heart failure.

The comparison between patients with and without sick euthyroid status revealed that those with SES tend to be older (59.90 years vs. 55.64 years), have higher pulse rates (111.81 bpm vs. 103.77 bpm), and have lower hemoglobin levels (10.658 g/dL vs. 11.144 g/dL). These findings suggest that patients with SES are generally in worse health, experiencing more severe symptoms and greater comorbidities. The elevated creatinine levels in the SES group (3.2677 mg/dL vs. 2.3903 mg/dL) indicate a potential link between renal impairment and thyroid dysfunction, supporting the hypothesis proposed by Klein and Ojamaa (2001) [21] that renal function can be adversely affected in patients with SES due to reduced cardiac output and associated systemic effects.

Therefore, this study provides valuable insights into the prevalence and impact of SES in chronic heart failure patients, with strengths including a comprehensive analysis of various clinical parameters, such as age, pulse rate, hemoglobin levels, jugular venous pressure, total T3, fasting total cholesterol, creatinine, NT-proBNP, LVEF, and Boston score. However, it has limitations, including a relatively small sample size, a single-center setting, and its cross-sectional design, which does not account for temporal changes. Additionally, the study did not include patients with sepsis, minors, or those with LVEF greater than 40% (heart failure with preserved ejection fraction, HFpEF). Further longitudinal studies are required to better understand patient outcomes over time.

Lastly, treating SES primarily involves addressing the underlying illness rather than directly correcting thyroid hormone abnormalities. SES itself does not typically require specific thyroid hormone replacement. The focus should be on managing critical conditions, such as heart failure, chronic kidney disease, or sepsis, which trigger the syndrome. Supportive care and optimizing overall patient health are essential [22]. In some cases, if there is a suspicion of underlying thyroid disease or if the patient does not recover as expected, further endocrine evaluation might be warranted.

## Conclusions

In conclusion, this study highlights the significant prevalence of SES among heart failure patients, with approximately two-fifths of patients affected. It underscores the intricate relationship between heart failure severity and thyroid function. The findings emphasize the necessity for regular thyroid function monitoring in heart failure patients to effectively identify and manage SES. Additionally, the observed correlations between NT-proBNP, total T3 levels, and heart failure severity underscore the importance of considering thyroid function as a critical factor in the comprehensive management of heart failure. Future research should focus on larger cohorts and explore the underlying mechanisms linking thyroid dysfunction to heart failure, potentially leading to improved therapeutic strategies.
